# Psychometric properties and longitudinal measurement invariance of the Brazilian version of the subjective happiness scale in adolescents

**Published:** 2021-03-16

**Authors:** Fernanda Ruffo Ortiz, Saul Martins Paiva, Isabela Almeida Pordeus, Thiago Machado Ardenghi

**Affiliations:** ^1^Department of Stomatology, Federal University of Santa Maria, Santa Maria, Brazil; ^2^Department of Pediatric Dentistry, Federal University of Minas Gerais, Belo Horizonte, Brazil

**Keywords:** adolescents, brazil, happiness, subjective well-being, validity

## Abstract

**Background:**

Happiness is a subjective construct. Validation studies to confirm validity and reliability of happiness measures are needed to verify its applicability in research and clinical fields.

**Aim:**

The aim of this study was to test the psychometric properties and longitudinal measurement invariance (MI) of the subjective happiness scale (SHS) in adolescents.

**Methods:**

A longitudinal study was conducted with a random sample of 1134 12-year-old adolescents from Santa Maria, a southern city in Brazil, starting in 2012. Two years later, 746 adolescents were reassessed, with an average age of 14. The Brazilian version of the SHS, which is composed of 4 items, was administered by a face-to-face interview. Reliability (Cronbach’s alpha), reproducibility (intraclass correlation coefficient – ICC), discriminant validity, confirmatory factor analysis (CFA), convergent validity, and MI were performed through the multigroup CFA. Socioeconomic, clinical, and subjective variables were also collected through clinical examinations and structured questionnaires by calibrated and trained dentists.

**Results:**

Cronbach’s alpha and ICC results were moderate (0.51 and 0.70, respectively). The scale was able to discriminate subjective happiness between different oral health groups and socioeconomic status. The CFA revealed a good fit model in both collections, confirming the validity of the scale. Convergent validity was satisfactory, demonstrating that the SHS is similar in theoretical concepts with a subjective scale. Moreover, MI showed a goodness-of-fit statistics across time points.

**Conclusion:**

The Brazilian version of SHS showed adequate validation properties and longitudinal measurement among adolescents.

**Relevance for patients:**

These findings are important for studies that evaluate happiness and oral disorders, through cross-section and longitudinal studies.

## 1. Introduction

Subjective assessments include psychological, social, emotional, and functional domains [1]. These depend on the individual’s self-perception, mood, and way of life and have been defined in the literature by the concepts of “subjective well-being,” “satisfaction with life,” and “happiness” [2,3]. Happiness has been defined as the degree to which individuals judge the overall quality of their life favorably [2]. It may be conceptualized as the product of a stable pattern of actions and reactions to life experiences, encompassing both emotional and cognitive domains. Still, the concept of happiness may vary between countries, cultures, and ages [3,4].

Some factors are directly linked to happiness, such as socioeconomic factors, values, age, mental, and physical health [2]. The previous studies have assessed the association of such predictors with positive well-being. The results demonstrated that healthy behaviors, higher socioeconomic status, fewer adverse activities, and better personal and social functions were associated with higher happiness levels [5-8]. Nevertheless, people who self-report as “unhealthy” also tend to feel less happy than their counterparts [7].

Subjective happiness is generally measured using self-report questionnaires that encompass either its affective or cognitive component [9]. Lyubomirsky and Lepper (1999) developed the subjective happiness scale (SHS) with the aim of providing an overall subjective measure to define whether one is a happy or unhappy person. This scale involves positive and negative aspects and encompasses affective and cognitive levels using 4 items. The scale was tested on American and Russian populations comprised different age groups (14–94 years old) and occupations. The reliability (internal consistency) and validation (construct validity) values were considered acceptable [10].

In Brazil, studies have demonstrated the validity of the Brazilian version of the SHS [11,12]. These studies provided important information for researchers, but the scale’s psychometric measures were applied to adult populations (mean age of 30 years). However, the scale was not assessed by longitudinal measurement. Longitudinal measurement invariance analyses showed whether the instrument parameters are equivalent or invariant among group and time points [13], being part of the multigroup confirmatory factor analysis (MGCFA). In this sense, happiness is a complex concept influenced by life circumstances and current feelings [7] and can show different patterns in adolescents. Therefore, the aim of this study was to test the psychometric properties and longitudinal measurement invariance of the SHS in Brazilian adolescents.

## 2. Methods

### 2.1. Study design and sample

We followed a longitudinal design with 12-year-old adolescents from Santa Maria, a southern city in Brazil. Adolescents were randomly selected in the city’s public schools. In stage sample, 20 out of 39 public schools [14] were enrolled, being equally distributed across the five administered regions of the city. Afterward, 12-year-old adolescents enrolled in these schools were invited to participate in the study. The total number of participants in 2012 was 1134.

**Figure 1:**
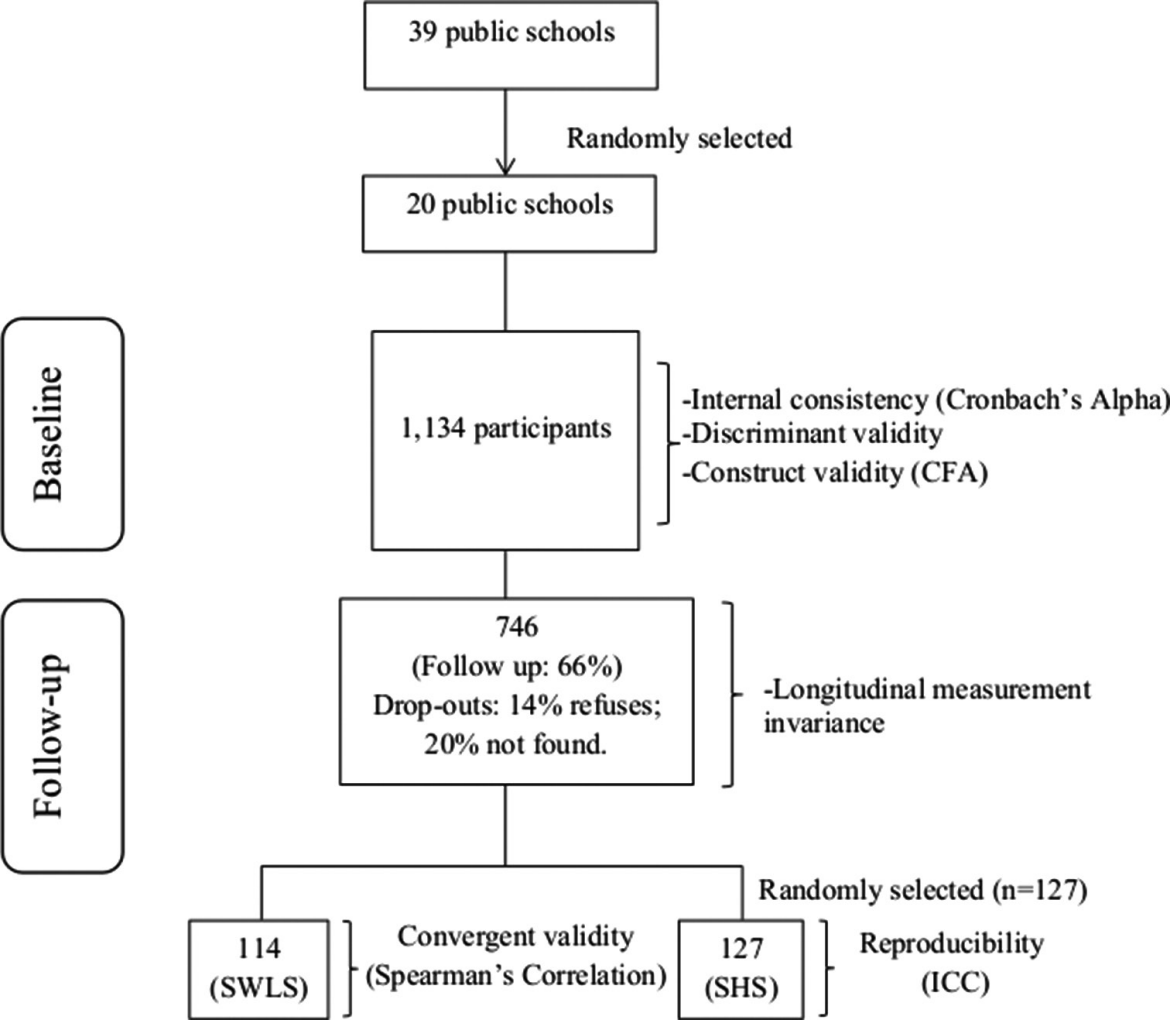
Distribution of the participants considered in each analysis.

In a second phase, the adolescents were reevaluated in 2014 to follow up the scale validation process. Seven hundred and forty-six adolescents with a mean age of 14 years were reassessed. A subset of 127 participants (17%) was randomly chosen to measure the test-retest reliability of the scale. This subset was contacted by one of the researchers and invited to take part in a subsequent study on happiness. The SHS was reapplied with a mean period of 2 weeks after the first application (Figure 1). This sample size was estimated using a minimum effect size to be detected of 0.3, 80% of power, 95% confidence interval, and 30% losses or refusals.

### 2.2. Data collection

The data collection process was assessed through clinical examinations and structured questionnaires. The two collections (2012 and 2014) were performed with the same methodological protocol. Data were conducted to measure the discriminant analysis.

Clinical examinations were performed in the schools by four calibrated dentists, following the criteria proposed by the World Health Organization [14]. The prevalence of dental caries was collected based on the decayed, missing, and filled teeth index for permanent teeth. The cavitated carious lesions variable was composed of the decayed component (D>0 component) of the index.

Socioeconomic characteristics were collected using a structured questionnaire sent to and answered by subjects’ parents. The variables collected were sex, household income, and household overcrowding. Household income was collected in Brazilian minimum wage (BMW), which corresponded to US $450 at the baseline. Analyses of household income were obtained from the median (1.6 BMW). Household overcrowding was calculated using the ratio of the number of rooms in a home to the number of people and was categorized as “1 room or more/person” or “less than 1 room/person.”

### 2.3. Subjective measures

The subjective measures included SHS, oral health-related quality of life (OHRQoL) questionnaire, and satisfaction with life scale (SWLS).

SHS was administered during face-to-face interviews with all participants. The SHS is a short form of 4 items developed by Lyubomirsky and Lepper (1999). The first two items of the scale are as follows: (SHS_a) “In general I consider myself,” and (SHS_b) “Compared to most of my friends, I consider myself.” Answers may range from 1 to 7, where 1 = “person considered less happy” and 7 = “person considered happier.” The other items are as follows: (SHS_c) “Some people are generally very happy. They enjoy life regardless of what is going on, getting the most out of everything. To what extent does this account describe you?” and (SHS_d) “Some people are generally not very happy. Although they are not depressed, they never seem as happy as they might be. To what extent does this characterization describe you?” Answers may range from 1 to 7, with 1 = “the sentence is nothing like the individual” and 7 = “the sentence is much like the individual.” For this last question, the response is encoded in reverse [10]. The SHS final score is the mean of the responses to the 4 items, with higher scores corresponding to higher happiness.

OHRQoL was measured using the Brazilian short version of the child perception questionnaire (CPQ11–14, ISF:16) [15], which was administered at the first phase. The questionnaire has 16 questions divided into four domains: Oral symptoms, functional limitation, emotional well-being, and social well-being. Answers may range from “never” to “every day” (0–4). Higher scores indicate worse OHRQoL.

The Brazilian version of the SWLS [16] was administered during face-to-face interviews in the second phase. The scale is composed by five questions to measure the dimensions of cognitive judgment and subjective well-being. The answers are given on a 7-point scale; higher scores indicate greater satisfaction with life.

### 2.4. Data analysis

Data analysis was performed using the software STATA 13.0 (Stata Corporation, College Station, TX, USA) and MPlus version 6.12.

The reliability and validity of the SHS were verified using different analyses to confirm its psychometric properties in adolescents. The internal consistency, discriminant, and construct validity were performed with the baseline participants. Reproducibility, convergent validity, and longitudinal measurement invariance were performed with the follow-up participants (Figure 1).

Cronbach’s alpha coefficient was used to assess the agreement between subsets of items. Reproducibility used the test-retest, calculated with the intraclass correlation coefficient (ICC). Values higher than 0.7 for alpha and ICC are considered acceptable [17]. The convergent validity of SHS was calculated through Spearman’s correlation coefficient, thus comparing the SHS’s theoretical concepts with the SWLS, assuming *P*<0.05.

Discriminant validity compared the mean scores of the SHS among socioeconomic, clinical, and subjective variables. The hypothesis was that subjects with socioeconomic disadvantages, dental caries, and worse OHRQoL would have lower happiness than their counterparts. This analysis took into account the sampling weight through the “svy” command. The effect size was also calculated to determine the magnitude of the mean differences between the predictors. The effect sizes were small (0.20), medium (0.50), and large (0.80) [18].

**Figure 2:**
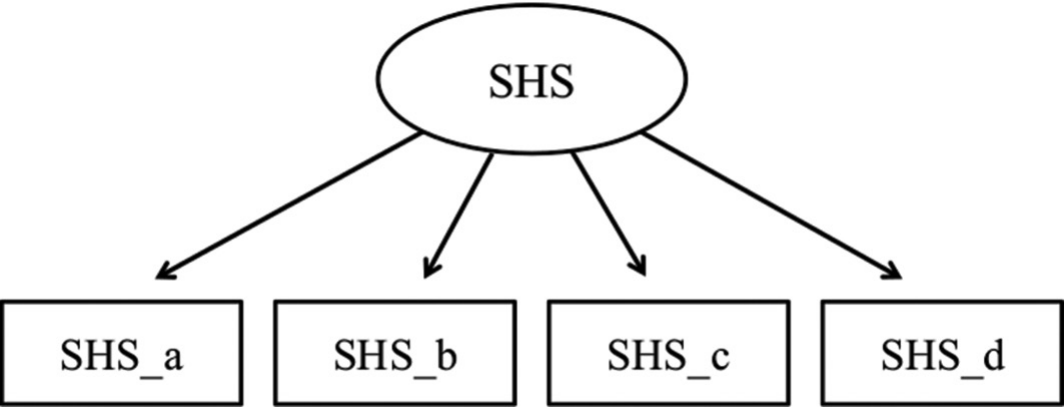
Model of the 4 items of SHS by confirmatory factorial analysis.

Construct validity of the SHS was assessed through confirmatory factorial analysis (CFA) with maximum likelihood estimation. The analysis evaluated the fit of the 4-item model (Figure 2) to the data on the sample in 2 times. The analyses of fit were performed with the aim of understanding the interaction among the 4 items, and whether they expressed the same theoretical concept of subjective happiness. The overall fit of the model was assessed based on the following parameters: Comparative fit index (CFI) and Tucker-Lewis index indicating any value above 0.95 as a good fit. Root mean square error of approximation (RMSEA) and standardized root mean square residual, with values of <0.07 and 0.08, respectively, were seen as a good fit [19].

Longitudinal measurement invariance analysis through MGCFA was also performed. This analysis allowed to compare the behavior of the SHS overtime and its equivalence across groups [13]. The analysis is divided into four levels: Configural, metric, scalar, and strict. Configural invariance assesses equivalence of the factor structure in the group, assuming the same loads on the factors; that is, if the different groups understand the same latent structure. Metric invariance assumes that the factor loadings are equivalent among groups. In this case, the weight of the loads must be equivalent. In scalar invariance, factor loading and item intercept need to be equal across groups. Strict invariance assumes that factor loading, item intercept, and residual variances are equal across groups. However, there is no consensus in the literature to evaluate strict invariance [20], making it optional. The global fit of the model is measured by CFI and RMSEA combined with variations among the models (D). The models should not show differences in the DCFI>0.01. The D involves comparing the fit between unconstrained and constrained models [21].

## 3. Results

The flowchart in Figure 1 shows the distribution of the participants considered in each analysis.

A total of 1134 adolescents with mean age of 12 years composed the first phase of the study. Most of the participants were female, had white skin color, belonged to families with low household income, and had parents with a higher educational level (≥8 years). The majority of the participants did not have cavitated carious lesions. In 2014, the characteristics were similar. The mean and standard deviation (SD) of happiness was 5.24 (SD 0.90) in 2012 and 5.38 (SD 0.90) in 2014 (Table 1).

**Table 1:** Descriptive analysis of the sample. Santa Maria, Brazil

Characteristic’s sample	2012	2014
	
	*n* (%)	*n* (%)
Sex		
Female	611 (53.9)	393 (52.7)
Male	523 (46.1)	352 (47.3)
Household income		
≤1.6 BMW	556 (53.7)	386 (69.1)
>1.6 BMW	480 (46.3)	173 (30.9)
Household overcrowding		
Less than 1 room/person	743 (68.7)	368 (60.4)
One room or more/person	339 (31.3)	252 (40.6)
Cavitated carious lesions		
Without	654 (57.7)	432 (58.1)
With	480 (42.3)	311 (41.9)
Continuous variables	Mean (SD)	Mean (SD)
SHS	5.24 (0.90)	5.38 (0.90)
CPQ 11–14	10.24 (7.59)	9.37 (7.31)
SWLS	-	5.32 (0.95)

BMW: Brazilian minimum wage (approximately U$ 450 during the data gathering).

SD: Standard deviation; CPQ 11–14: Child perception questionnaire; SHS: Subjective happiness scale; SWLS: Satisfaction with life scale

Table 2 displays the descriptive distribution of the SHS items scores. Score 7 was the most frequently reported in SHS_a item, revealing that adolescents considered themselves happier. In SHS_b and SHS_c, there was a balance between scores 4 and 7. The highest mean was observed for the SHS_a (5.91, SD 1.22) and the lowest mean for the SHS_d (4.36, SD 1.86).

**Table 2:** Descriptive distribution of SHS items scores (n 1134). Santa Maria, Brazil

Items	Scores							Mean (SD)

	1	2	3	4	5	6	7	
						
	*n* (%)	*n* (%)	*n* (%)	*n* (%)	*n* (%)	*n* (%)	*n* (%)	
SHS_a – “In general, I consider myself a very happy person”	9 (0.9)	13 (1.2)	18 (1.6)	102 (8.9)	220 (19.4)	293 (25.8)	479 (42.2)	5.91 (1.22)
SHS_b – “Compared to most of my peers, I consider myself”	19 (1.7)	20 (1.8)	56 (4.9)	193 (17.0)	214 (18.9)	313 (27.6)	319 (28.1)	5.45 (1.41)
SHS_c – “Some people are generally very happy. They enjoy”	39 (3.4)	39 (3.4)	68 (6.0)	169 (14.9)	264 (23.3)	280 (24.7)	275 (24.3)	5.22 (1.55)
SHS_d – “Some people are generally not very happy. Although”	78 (6.9)	122 (10.7)	206 (18.2)	203 (17.9)	168 (14.8)	142 (12.5)	215 (19.0)	4.36 (1.86)
Total SHS								5.24 (0.90)

SD: Standard deviation; SHS: Subjective happiness scale

The internal consistency and reproducibility of the SHS were moderate; Cronbach’s alpha value was 0.51 and ICC value was 0.70 (95% CI: 0.60–0.77). There was a significant correlation between the SHS and SWLS, confirming their convergent validity, whereas Spearman’s correlation coefficient was 0.35, with *P*<0.01 (data not reported in tables).

The discriminant validity of the SHS mean scores according to the different predictors is shown in Table 3. The questionnaire was able to discriminate subjective happiness between socioeconomic status and oral conditions. Children with low socioeconomic status, dental caries, and poor OHRQoL presented lower levels of happiness than their counterparts.

**Table 3:** Descriptive values of discrimination validity to sample (*n*: 1134 in 2012 and *n*: 746 in 2014). Santa Maria, Brazil

	Mean SHS score* (SE) 2012	P**	Change scores (SD) 2012	Mean SHS score* (SE) 2014	P**	Change scores (SD) 2014	Effect size
Household income						
>1.6 BMW***	5.35 (0.06)	0.000	0.05 (0.82)	5.36 (0.07)	0.014	0.11 (0.81)	0.14
≤1.6 BMW***	5.15 (0.05)		0.16 (0.87)	5.16 (0.06)		0.11 (0.81)	0.17
Household overcrowding						
One room or more/person	5.32 (0.04)	0.000	0.03 (0.82)	5.31 (0.05)	0.004	0.08 (0.88)	0.20
Less than 1 room/person	5.05 (0.06)		0.10 (0.88)	5.08 (0.08)		0.14 (0.82)	0.37
Cavitated carious lesion							
Without	5.29 (0.05)	0.048	0.08 (0.82)	5.31 (0.06)	0.015	0.03 (0.82)	0.33
With	5.16 (0.04)		0.11 (0.90)	5.15 (0.06)		0.23 (0.92)	0.17
CPQ 11–14							
Without	5.37 (0.05)	0.000		5.38 (0.06)	0.000		0.17
With	5.07 (0.04)			5.08 (0.06)			0.17

* Taking into account the sampling weight.

** Mann–Whitney U-test. SE: Standard error, SD: Standard deviation, SHS: Subjective happiness scale; BMW: Brazilian minimum wage (approximately U$ 450 during the data gathering), CPQ: Child perception questionnaire

The internal consistency results of CFA are displayed in Table 4. In 2012, the latent variable (SHS) was statistically related to the first 3 items (SHS_a, SHS_b, and SHS_c). In contrast, the SHS_d item had a low factor load and was not statistically associated with happiness. In 2014, the latent variable (SHS) was statistically related to the four items. Table 5 shows MGCFA through longitudinal measurement invariance. The model comparisons indicate the goodness-of-fit statistics for tests of measurement invariance across time points.

**Table 4:** Measures of internal consistency of the confirmatory factorial analysis for the SHS model in 2014

	Items	Standardization load	Residual variances	*P*-value	Reliability
2012					
	SHS_a	0.666	0.556	0.000	0.444
	SHS_b	0.507	0.743	0.000	0.257
	SHS_c	0.350	0.877	0.000	0.123
	SHS_d	0.038	0.999	0.358	0.001
2014					
	SHS_a	0.885	0.218	0.000	0.782
	SHS_b	0.539	0.710	0.000	0.290
	SHS_c	0.415	0.827	0.000	0.173
	SHS_d	0.187	0.965	0.000	0.035

Reliability is the square of the standardized load, SHS: Subjective happiness scale

**Table 5:** Goodness-of-fit statistics for measurement invariance across time points

Model	CFI	RMSEA	DCFI	DRMSEA
Invariance across T1 and T2				
Configural	0.978	0.023		
Metric	0.974	0.026	0.004	0.003
Scalar	0.976	0.024	0.002	0.001

T1:2012, T2:2014. CFI: Comparative fit index, RMSEA: Root mean square error of approximation. Δ: Combined variations among the models

## 4. Discussion

This study evaluated the psychometric properties and longitudinal measurement of the SHS in adolescents. In general, the results indicated that the SHS is valid for measuring subjective happiness in Brazilian adolescents, including longitudinal validity across time points. The result was demonstrated by discriminant and convergent validity, CFA, and measurement invariance.

In this study, Cronbach’s alpha showed a low and not acceptable value. There is critical literature regarding the use of this measure to assess data reliability and internal insistence [22]. Hence, the results should be interpreted with some caution. Instruments with lower numbers of items tend to have lower alpha values [23]. The argument has been made that a single test administration does not allow for the precision of individual test performance [24]. Nevertheless, lower Cronbach’s alpha values can be regarded and accepted, as the test is short and low reliability levels would be expected. The coefficient itself cannot be interpreted as a measure of internal consistency [24], and study has suggested the use of a more robust analysis, such as the CFA [25].

The reproducibility value was acceptable, showing a correlation when the SHS was reapplied. Low values for reproducibility have been reported when psychological measurements were applied due to bias and artifacts that are inherent to these scales [9]. The variations in responses are considered normal because they depend on each individual [26,27], even more so as adolescence can be a phase of constant change. However, we cannot deny that the results presented limit values when the scale was applied overtime. One possible explanation is that the issues were poorly understood, and individuals could have been confused when they were answering. The possibility of a response shift between the first and second administrations cannot be ruled out. The reproducibility values may also have been affected by the inconsistency of the SHS_d item or by the complexity of subjective measurement. Furthermore, adolescence is a constant transformation phase, in which circumstances can act in daily life, and in a period of weeks or days, adolescents may change their perception of happiness.

The theoretical similarity between the SHS and SWLS was confirmed by the convergent validity results. These scales demonstrated a statistically significant correlation (*P*<0.01). Concepts of happiness and satisfaction with life are considered synonyms, representing a similar theoretical direction [2], which was confirmed by our results. Still, subjective definitions depend on the individual’s perception of their mood and way of life being related to well-being, where favorable events tend to indicate satisfaction and happier people [2].

The discriminant validity analysis allowed for comparing socioeconomic groups, clinical and subjective variables with the SHS mean. The interpretation of low, medium, or high effect size values is references resulting from a convention [17]. Moreover, a difference corresponds to an impact that must be taken into account, even if it is small for subjective outcomes. Children with low socioeconomic status, poor oral health, and poor OHRQoL showed lower levels of happiness. Studies have reported that socioeconomic disadvantages have an impact on individuals’ psychological well-being [27,28]. Socioeconomic disadvantages influence oral health outcomes through different pathways [29]; they may lead to the low accumulation of resources and knowledge, which limits the adoption of healthy habits and decision-making [30]. Another noteworthy fact is that deprivation may affect how people feel and rate their health in comparison with people on the same social level for psychosocial reasons [31]. Moreover, children and adolescents with oral disorders tend to experience more dental discomfort and functional limitations [28]. They are likely to feel upset and concerned about their health, affecting the emotional and social domains [28]. These factors impair the OHRQoL and, as a consequence, their happiness. Once happiness is affected by socioeconomic, clinical, and subjective variables, national public policies may be idealized to provide an improvement in the well-being of individuals [9].

The CFA allowed for verification of the relationships between the items of the SHS and for ascertaining if they expressed the same meaning of happiness. The global model presented a quality of fit that was good and acceptable [25] when administered to the 12- and 14-year-old adolescents. The CFA showed statistically significant relationships between the items, except for SHS_d in 2012. This exception may have been due to the lack of understanding of this item by the participants or by the fact that the item has inverse sense when compared to the others. Another possible explanation is the lack of cross-cultural validation or even by the scale not being made for such young people. Furthermore, this item (SHS_d) had already presented problems in previous publications [11]. On the other hand, the results performed in 2014 were acceptable. It may be justified by a greater maturity of the adolescents.

Longitudinal studies have used subjective measure, showing longitudinal measurement invariance for OHRQoL [32] and SWLS [33]. Hence, examining longitudinal invariance of SHS is also relevant. The measurement invariance showed that overtime (2 years) SHS was equivalent across factor loading and intercept for items. The result indicates that happiness can be assessed in longitudinal studies among adolescents.

This study has some limitations, as it did not evaluate the cross-cultural and semantic validation of the SHS. There are no reports in the literature on this type of validation for this scale. Thus, we believe that a qualitative study, with basic theoretical transcultural principles, should be carried out with measurements of happiness.

## 5. Conclusion

The Brazilian version of the SHS showed adequate validation properties and longitudinal measurement invariance in a population of adolescents. This paper is important for studies that aim to evaluate happiness and oral disorders through cross-section and longitudinal studies. Decisions related to public policies can be carried out based on the subjective and normative knowledge of the health condition of a specific population.
